# Supplementary material for: Quantifying nitrogen fixation by heterotrophic bacteria in sinking marine particles
Source: Nat Commun. 2021 Jul 2;12:4085. doi: 10.1038/s41467-021-23875-6 (PMC8253789; doi:10.1038/s41467-021-23875-6)
Supplement: Supplementary file 1 — Supplementary file [file 41467_2021_23875_MOESM1_ESM.pdf]

Nature Communications

Supplementary Information for

Quantifying nitrogen fixation by heterotrophic bacteria in sinking marine particles

Subhendu Chakraborty[1,2,*], Ken H. Andersen[2], André W. Visser[2], Keisuke Inomura[3], Michael J. Follows[4], Lasse Riemann[1,*]

[1]*Department of Biology, Marine Biological Section, University of Copenhagen, Helsingør, Denmark*

[2]*Centre for Ocean Life, DTU Aqua, Technical University of Denmark, Kemitorvet, 2800 Kgs. Lyngby, Denmark*

[3]*School of Oceanography, University of Washington, Seattle, WA, USA*

[4]*Department of Earth, Atmosphere and Planetary Sciences, MIT, Cambridge, MA, United States of America*

Current address (Subhendu Chakraborty): *Systems Ecology Group, Leibniz Centre for Tropical Marine Research (ZMT), Bremen, Germany*

[*]Corresponding authors: Department of Biology, Marine Biological Section, University of Copenhagen, Helsingør, Denmark. Tel: +4591714664. Email: schakraborty@bio.ku.dk; lriemann@bio.ku.dk

The material provides detailed information about the sources and ways of choosing parameters, and some additional figures for a better understanding of the N_2 fixation and dynamics inside sinking particles.

S1. Model parameterization

Parameters originate from literature sources and were converted into appropriate units as needed. Concentrations inside the particle are calculated as per liter of particle and outside as per liter of water.

Initial concentrations:

As initial concentrations of polysaccharide and polypeptide, we applied 20% of the concentrations measured in aggregates (polysaccharides and combined amino acids, respectively) generated artificially using rolling tanks from artificial seawater amended with phytoplanktonic material[1]. This was done because laboratory-made particles are richer than naturally occurring particles[2]. The labile fraction of carbohydrate is found to vary between 1.4 –64.6% with an average of 23.8% in the adjacent open slope of Blanes canyon (NW Mediterranean Sea) and here we consider a similar fraction 23.8% of polysaccharides as labile[3]. The labile fraction of proteins varies from 1.4 – 97.4% in waters of different trophic status[4]. We chose that 50% of polypeptides are labile. To convert polysaccharide concentration into glucose units, we assume that glucose is the dominant neutral sugar in polysaccharides and that these contain 53% combined glucose[5]. Molecular weights of 180 g $mole^{-1}$ and 120.5 g $mole^{-1}$ are used for glucose and amino acids, respectively, to convert them into μg L^{-1} unit.

Most studies provide bacterial abundance as the number of cells per liter of water; e.g. bacterial density on transparent exopolymer particles was $\sim 10^9 - 10^{10}$ cells per liter water[6,7]. However, the number of cells per liter of particle is much greater than the number of cells per liter water; e.g. 2×10^{12} cells per liter of artificially produced aggregate[1]. In our study, we assume 10^{10} cells per liter of particle as the initial concentration of bacteria.

Initial concentrations of glucose, amino acids, O_2, ${NO_3}^-$, and ${SO_4}^{2-}$ within particles is assumed to be similar to concentrations in the surrounding water. For glucose, amino acids, ${NO_3}^-$,

and SO_4^{2-}, these are 50 μg L^{-1} [8], 5 μg L^{-1} [8], 10 μmol L^{-1} [9], and 29×10^3 μmol L^{-1} [10], respectively. To investigate the mechanisms of N_2 fixation, O_2 concentration is chosen 50 μmol L^{-1} from the range where laboratory experiments confirmed N_2 fixation in heterotrophic bacteria[11]. Since O_2 concentration in the world ocean varies within the range 0-400 μmol L^{-1} [12], we vary O_2 concentration within this range to calculate the maximum possible range of O_2 for the occurrence of N_2 fixation in heterotrophic bacteria.

C and N content in G and A:

The fraction of C in G ($f_{\mathrm{G,C}}$) is 0.4 and the fractions of C ($f_{\mathrm{A,C}}$) and N ($f_{\mathrm{A,N}}$) in A are assumed to be 0.445 and 0.125, respectively[13].

Parameters related to cell structure:

We calculate the cell radius (r_{B}) by using the volume (V_{B} μm^3) to C content (x_{B} fg C cell^{-1}) relationship from[14]

$$x_{\mathrm{B}} = 133.754 \times V_{\mathrm{B}}^{0.438}. \quad \text{(S1)}$$

By choosing $x_{\mathrm{B}} = 50$ fg C cell^{-1} for a particle-associated bacterium[15], we get $r_{\mathrm{B}} = 0.29$ μm. Of this radius, we choose a thickness of the cell wall of 10 nm[16] and of the cell membrane layer or plasma membrane (L_{m}) of 6 nm[16], both values originate from Gram-negative bacteria. The radius of the cellular cytoplasm (r_{C}) then equals 0.27 μm.

Parameters related to hydrolysis, uptakes, and N_2 fixation rate:

The maximum exoenzymatic hydrolysis rates are taken from[17]. Their maximum α-glucosidase and aminopeptidase activities are converted to obtain the maximum hydrolysis rate of polysaccharide, $h_{\mathrm{C}} = 2.25 \times 10^{-5}$ μg G cell^{-1} d^{-1}, and maximum hydrolysis rate of polypeptide, $h_{\mathrm{P}} = 6.1 \times 10^{-5}$ μg A cell^{-1} d^{-1}, respectively. Half-saturation constants of hydrolysis by β-glucosidase and aminopeptidas[18] are converted to obtain half-saturation constants of polysaccharide and polypeptide hydrolysis as 1.8×10^4 μg G L^{-1} and 3.6×10^3 μg A L^{-1}, respectively. Then by dividing maximum hydrolysis rates with half-saturation constants, we get corresponding affinities A_{C} and A_{P}.

The maximum glucose uptake rate and half-saturation constant of uptake are taken from[1]. We converted C units into glucose units by using the factor $f_{\mathrm{G,C}}$ and then divided the maximum glucose

uptake rate by the half-saturation constant to obtain glucose affinity. Similarly, we obtain the maximum amino acid uptake rate and the corresponding affinity from[19].

For the maximum NO_3^- uptake rate, we use a NO_3^- uptake rate estimated for free-living marine bacteria[20]. We use total NO_3^- uptake and bacterial abundance from their station 43 to calculate maximum NO_3^- uptake rate per cell as 1.63×10^{-9} µmol NO_3 $cell^{-1}$ d^{-1}. Regarding NO_3^- affinity, we use a cell-specific ammonia affinity for marine bacteria[21].

Maximum SO_4^{2-} uptake rate of 5×10^{-10} µmol SO_4 $cell^{-1}$ d^{-1} is taken from a range of cell-specific SO_4^{2-} reduction rate for bacteria in a river sediment[22].

For the marine heterotrophic diazotroph *Rhodopseudomonas palustris* BAL398, Bentzon-Tilia et al.[23] measured the highest cell-specific C_2H_4 production rate of 0.515 fmol C_2H_4 $cell^{-1}$ h^{-1}. Using the conversion ratio 3:1 of ethylene to N[24], we obtain a maximum N_2 fixation rate of $M_{N_2} =$ 5.77 µg N $cell^{-1}$ d^{-1}.

Parameters related to cost:

The cost of enzyme production ($R_E = 0.6$ µg C µg C^{-1}) and the basal respiratory cost ($R_B = 0.05$ µg C µg C^{-1}) are taken from a theoretical study investigating strategies used by particle-attached bacteria to counteract diffusive losses of exoenzymes and hydrolysate in a water column[25]. For the cost of amino acids uptake ($R_A = 0.23$ µg C µg C^{-1}), we use the respiratory cost of bacterial assimilation of amino acids[26]. The cost of glucose uptake (R_G) is assumed identical to the cost of amino acid uptake. For the cost of NO_3^- uptake, the respiratory cost for bacterial assimilation of dissolved inorganic N is used ($R_{NO_3} = 0.4$ µg C µg C^{-1} [26]). To make SO_4^{2-} energetically less favorable, we chose a larger value of the cost of SO_4^{2-} uptake compared to the uptake cost of NO_3^- ($R_{SO_4} = 0.6$ µg C µg C^{-1}).

Regarding the direct respiratory cost of N_2 fixation, 2.04 moles carbohydrate is needed to produce one mole of NH_3[27], which yields $R_{N_2} = 0.4$ µg C µg C^{-1}. The indirect cost of N_2 fixation in terms of O_2 removal from the cell (R_{O_2}) can be obtained by converting the diffusive flux of O_2 in the cell into C equivalents (Eq. 19).

Parameters related to the particle:

We consider a particle size range of 10 μm – 5 mm in diameter representative of the open ocean[28].

Parameters related to diffusion:

We assume a similar diffusion coefficient of amino acid inside a particle as that of glucose (D_{M})[29].

Direct measurement of apparent diffusivity of O_2 within diatom aggregates using a diffusivity microsensor shows values of 0.90 to 0.95 times the free diffusion coefficient in seawater[30]. In the present study, the diffusion coefficients of O_2 and NO_3^- inside particles ($\overline{D}_{O_2}, \overline{D}_{NO_3}$) are assumed 0.95 times that of the free diffusion coefficient in seawater (D_{O_2}, D_{NO_3}). Since, compact particles, like fecal pellets, can have less diffusivity inside particles, we performed a sensitivity analysis to examine how changes in apparent diffusivity (f_{O_2}) affect N_2 fixation in particles. This suggests that N_2 fixation rate will increase in compact particles with lower apparent diffusivity (Fig. S2).

Moreover, diffusion of O_2 and NO_3^- into a cell is restricted by the cell membrane layers. We use the value $\varepsilon_{\mathrm{m}} = 7.9 \times 10^{-4}$ for the diffusivity of cell membrane layers relative to water[31]. Using these values, the effective diffusion coefficient of O_2 due to cell membrane layers (K_{O_2}) and diffusive O_2 inflow into the cell (F_{O_2}) can be calculated (see Eq. (16) and (15) in the main text).

Finding optimal N_2 fixation rate

Bacterial cells optimize their N_2 fixation rate to yield the highest population growth rate. To avoid making the optimization in Eq. (30) at every time step during the simulation, a lookup table of the parameter $\psi(G, A, X_{O_2}, X_{NO_3})$ determining N_2 fixation over possible realistic ranges of four environmental factors C, N, O_2, and NO_3^- is created at the beginning of the simulation. Specifically, all environmental factors are discretized into 40 logarithmically spaced intervals over their realistic ranges.

Sensitivity analysis

The sensitivity of the N_2 fixation rate per particle (J_{N_2}) is examined by varying some of the parameters by ±25% (Fig. S2) from their default values (Table S1). Specifically, we examine the

sensitivity of the parameters related to costs (R_{B}, R_{E}, R_{G}, R_{A}, $R_{\mathrm{N_2}}$, $R_{\mathrm{NO_3}}$, $R_{\mathrm{SO_4}}$), hydrolysis rates and maximum uptake rates (h_{C}, h_{P}, M_{G}, M_{A}, $M_{\mathrm{NO_3}}$, $M_{\mathrm{SO_4}}$), fraction of $\mathrm{O_2}$ diffusivity within particle compared to water ($f_{\mathrm{O_2}}$), bacterial C:N ($\rho_{\mathrm{CN,B}}$), and diffusivity of cell membrane layers relative to water (ε_{m}). The sensitivity index (I) is defined as $I = (\overline{J_{\mathrm{N_2}}} - J_{\mathrm{N_2}}) \times 100/J_{\mathrm{N_2}}$, where $J_{\mathrm{N_2}}$ is the value of the variable with reference parameters mentioned in Table S1 and $\overline{J_{\mathrm{N_2}}}$ is the value of the $\mathrm{N_2}$ fixation rate obtained when the parameter is varied by ±25%. This measure gives the relative change of $J_{\mathrm{N_2}}$. A few parameters strongly influence the $\mathrm{N_2}$ fixation rate. The most important parameters are maximum hydrolysis rate of polysaccharide (h_{C}), cost of amino acids uptake (R_{A}), maximum glucose uptake rate (M_{G}), maximum amino acid uptake rate (M_{A}), and the fraction of $\mathrm{O_2}$ diffusivity within particle compared to water ($f_{\mathrm{O_2}}$).

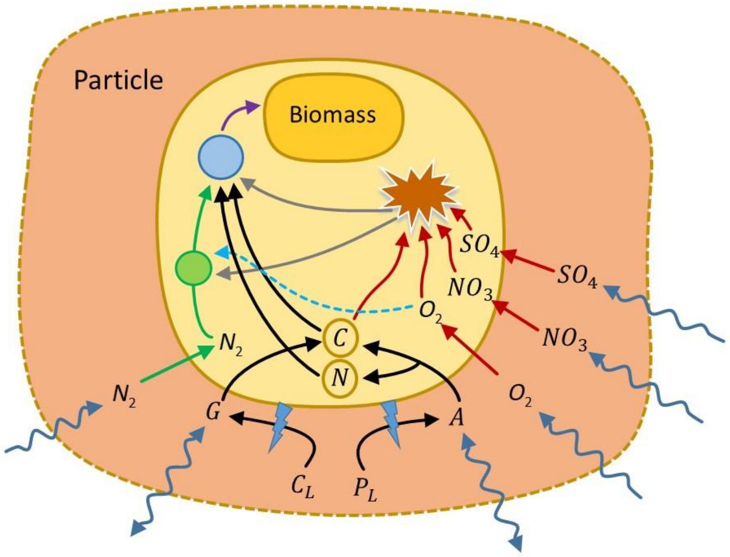

Fig. S1 Schematic diagram representing the interaction between a cell (yellow), a particle (orange), and the surrounding environment. Wavy lines represent diffusive exchange of molecular oxygen (O_2), nitrate (NO_3^{-}), sulfate (SO_4^{2-}), molecular nitrogen (N_2), glucose (G), and amino acids (A) between the particle and the surrounding environment. Light blue lightning symbols represent hydrolysis of polysaccharides (C_L) and polypeptides (P_L) into glucose and amino acids. Black arrows represent the pathway for obtaining carbon (C) and nitrogen (N) for biomass synthesis (blue circle). Green arrows represent N_2 fixing pathway where N_2 is converted into NH_4 (green circle) and provide additional N to the cell. Cyan dashed arrow is the regulation of N_2 fixation depending on the cellular O_2 concentration. Red arrows represent the energy production pathways through respiration (brown explosion symbol), gray arrows represent the regulation of N_2 fixation and biomass synthesis by generated ATP that is not modeled and not exclusively mentioned in the diagram, and finally, the magenta arrow represents biomass generation.

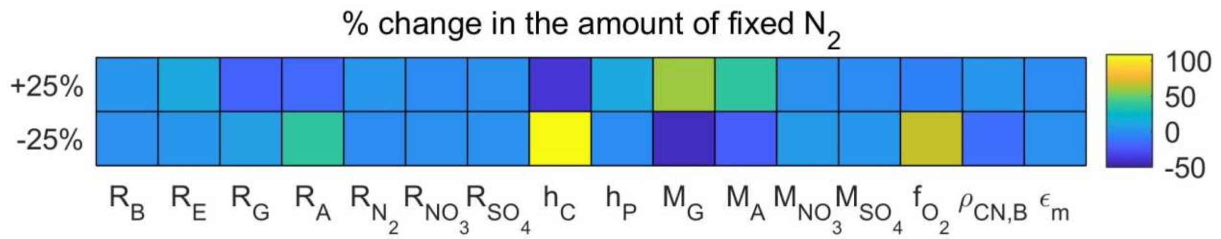

Fig. S2 Sensitivity analysis of N_2 fixation rates per particle by changing default values of the parameters in the supplementary Table S1 with ±25%. Sensitivities are expressed as percentages, represented by a colour according to the scale on the right. The 25% increased value of f_{O_2} is set to its maximum value 1.

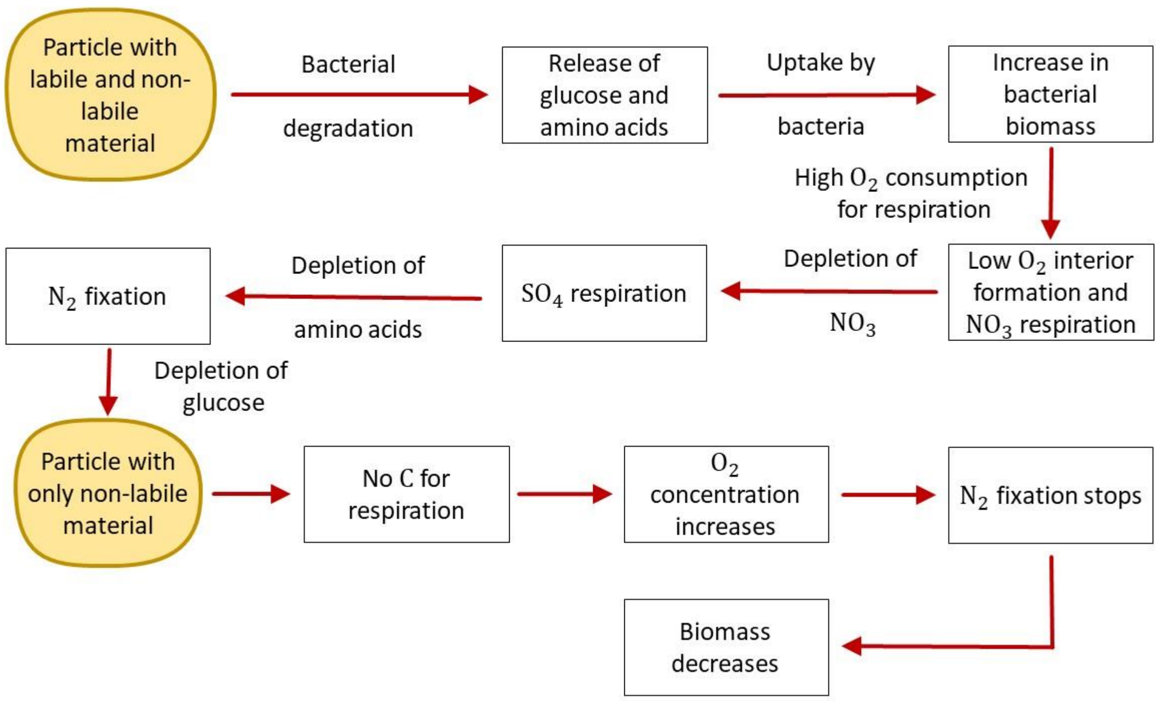

Fig. S3 A conceptual flow chart of how different factors are responsible for different events inside particles.

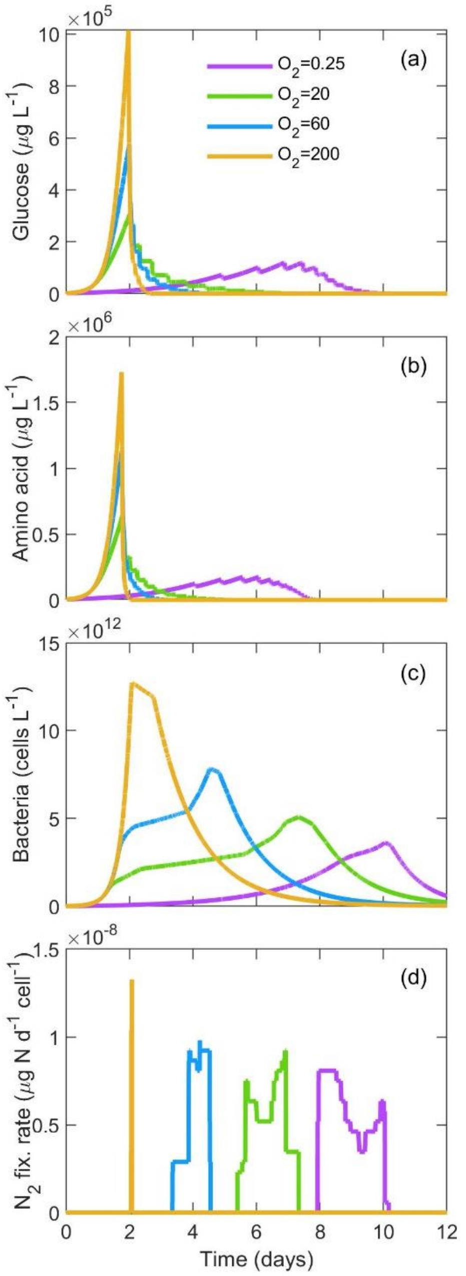

Fig. S4 Comparison of temporal variations of glucose concentrations (a), amino acid concentrations (b), bacterial abundances (c), and N_2 fixation rates (d) at four different environmental O_2 concentrations (0.25, 20, 60, and 200 μmol O_2 L^{-1}) at a radial distance of 0.21 cm from the center of the particle of radius 0.25 cm. Parameter values and environmental conditions are same as in Fig. 3.

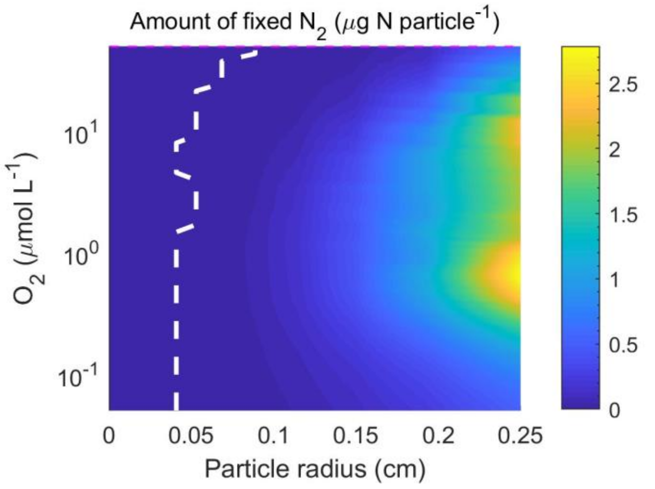

Fig. S5 The total amount of fixed N_2 per particle as a function of particle radius and environmental O_2 concentrations. The regions of occurrence ($N_{fix} > 10^{-3}$ μg N particle^{-1}) and non-occurrence of N_2 fixation are separated by the white dashed line. Initial labile polysaccharides and polypeptides concentrations are $C_L = 2 \times 10^7$ μg L^{-1} and $P_L = 2 \times 10^7$ μg L^{-1}, respectively. Parameter values are taken from Table S1.

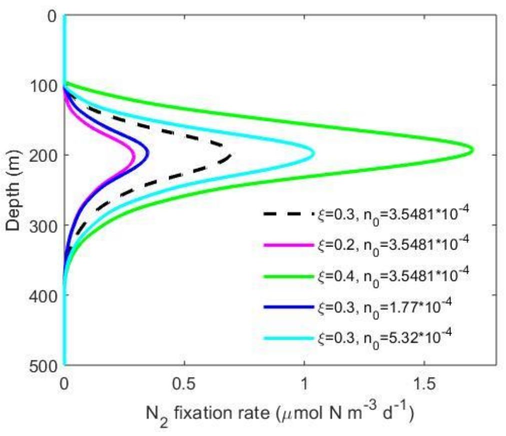

Fig. S6 Sensitivity test of N_2 fixation rate per unit volume of water. The proportion of large and small particles (ξ; 0.2 and 0.4) and the parameter determining the abundance of particles by ±50% (n_0; 1.77×10^{-4} and 5.32×10^{-4}) from its default value (3.5481×10^{-4}) are varied. The dashed curve represents the N_2 fixation rate at the default value (ξ=0.3, n_0=3.5481×10^{-4})[37]. Here the sinking speed of particles is similar to natural marine snow and vertical O_2 and NO_3^- concentrations are taken from the Mauritanian upwelling zone in the North Atlantic Ocean (NAO)[9].

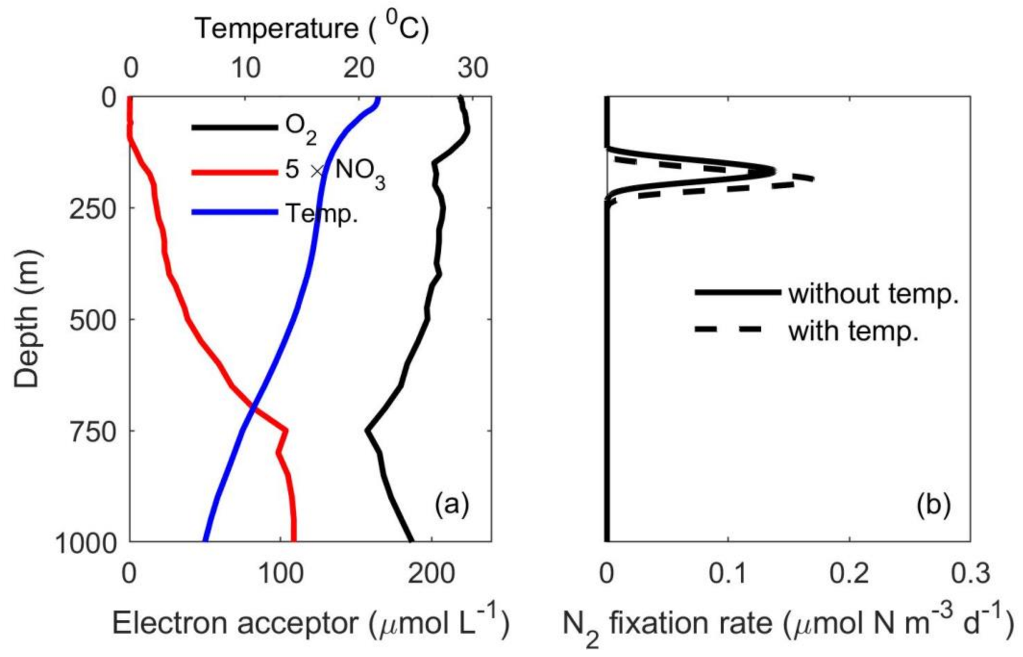

Fig. S7 Comparison of predicted N_2 fixation rate in natural marine snow in an open ocean site in the North Atlantic Ocean (30.5°N, 52.5°W)[32,33] in the presence and absence of temperature regulation. (a) O_2, NO_3^-, and temperature profiles in the water column[9]. (b) N_2 fixation rates per unit volume of water in the presence and absence of temperature regulation.

Table S1. Initial concentrations of variables and parameter values. Everything inside the particle is calculated as per liter of particle and outside as per liter of water. G and A indicate glucose and amino acids, respectively.

Symbol	Description	Initial conc./ Parameter value	Unit	Source
C_P	Initial polymeric polysaccharide	2.6×10^8	µg G L^{-1}	[1]
P_P	Initial polymeric peptide	1.6×10^8	µg A L^{-1}	[1]
f_C	Labile fraction of C_P	0.238	–	[3]
f_P	Labile fraction of P_P	0.5	–	[3]
C_L	Labile C_P	$f_C C_P$	µg G L^{-1}	–
P_L	Labile P_P	$f_P P_P$	µg A L^{-1}	–
G_∞	Glucose conc. outside particle	50	µg L^{-1}	[8]
A_∞	Amino acids conc. outside particle	5	µg L^{-1}	[38]
$X_{O_2,\infty}$	O_2 conc. outside particle	50	µmol O_2 L^{-1}	[11]
$X_{NO_3,\infty}$	NO_3^- conc. outside particle	15	µmol NO_3 L^{-1}	[9]
$X_{SO_4,\infty}$	SO_4^{2-} conc. outside particle	28×10^3	µmol SO_4 L^{-1}	[39]
G	Initial glucose conc. in particle	G_∞	µg L^{-1}	–
A	Initial amino acids conc. in particle	A_∞	µg L^{-1}	–
X_{O_2}	Initial O_2 conc. in particle	$X_{O_2,\infty}$	Mmol O_2 L^{-1}	–
$X_{O_2,c}$	Initial O_2 conc. in cell	$X_{O_2,\infty}$	µmol O_2 L^{-1}	–
X_{NO_3}	Initial NO_3^- conc. in particle	$X_{NO_3,\infty}$	µmol NO_3 L^{-1}	–

X_{SO_4}	Initial SO_4^{2-} conc. in particle	$X_{SO_4,\infty}$	mmol SO_4 L^{-1}	–
B	Initial bacteria conc. in particle	10^{10}	cells L^{-1}	–
h_C	Max. hydration rate of C_P	2.25×10^{-6}	μg $cell^{-1}$ d^{-1}	40
A_C	Affinity of C_P hydration	9.0×10^{-9}	L $cell^{-1}$ d^{-1}	40
h_P	Max. hydration rate of P_P	1.5×10^{-6}	μg $cell^{-1}$ d^{-1}	40
A_P	Affinity of P_P hydration	8.96×10^{-9}	L $cell^{-1}$ d^{-1}	40
M_G	Max. G uptake rate	7.0×10^{-7}	μg $cell^{-1}$ d^{-1}	1
A_G	Affinity for G uptake	2.77×10^{-9}	L $cell^{-1}$ d^{-1}	1
M_A	Max. A uptake rate	4.42×10^{-7}	μg $cell^{-1}$ d^{-1}	1
A_A	Affinity for A uptake	6.95×10^{-9}	L $cell^{-1}$ d^{-1}	1
M_{NO_3}	Max. NO_3^- uptake rate	1.63×10^{-9}	μmol NO_3 $cell^{-1}$ d^{-1}	20
A_{NO_3}	Affinity for NO_3^- uptake	4.26×10^{-8}	L $cell^{-1}$ d^{-1}	41
M_{SO_4}	Max. SO_4^{2-} uptake rate	5×10^{-10}	μmol SO_4 $cell^{-1}$ d^{-1}	22
$f_{G,C}$	Fraction of C in G	0.4	–	3
$f_{A,C}$	Fraction of C in A	0.445	–	38
$f_{A,N}$	Fraction of N in A	0.125	–	38
M_{N_2}	Max. N_2 fixation rate	5.77×10^{-8}	μg N $cell^{-1}$ d^{-1}	23
Ψ	Parameter determining N_2 fixation	Variable ($0 < \Psi < 1$)	–	–
R_B	Basal maintenance cost	0.05	d^{-1}	25
R_E	Cost of exoenzyme prod.	0.6	d^{-1}	25
R_G	Direct cost of G uptake	0.2	μg C μg C^{-1}	26

R_A	Direct cost of A uptake	0.23	μg C μg C^{-1}	[26]
R_{N_2}	Direct cost of N_2 fixation	0.4	μg C μg C^{-1}	[27]
R_{NO_3}	Direct cost of $NO_3{}^-$ uptake	0.4	μg C μg C^{-1}	Assumed
R_{SO_4}	Direct cost of $SO_4{}^{2-}$ uptake	0.6	μg C μg C^{-1}	Assumed
ρ_{CO}	Conversion of respiratory O_2 to C equivalents	10	μg C (μmol O_2)$^{-1}$	[17]
ρ_{CNO_3}	Conversion of respiratory $NO_3{}^-$ to C equivalents	12.5	μg C (μmol NO_3)$^{-1}$	[42]
ρ_{CSO_4}	Conversion of respiratory $SO_4{}^{2-}$ to C equivalents	20	μg C (μmol SO_4)$^{-1}$	[43]
f_{O_2}	Fraction of O_2 diffusivity within particle compared to water	0.95	–	[30]
D_{O_2}	Diffusion coefficient of O_2 in water	2.12×10^{-5}	cm^2 s^{-1}	[44]
$\overline{D}_{O_2}$	Diffusion coefficient of O_2 inside particles	$f_{O_2} \times D_{O_2}$	cm^2 s^{-1}	–
D_{NO_3}	Diffusion coefficient of $NO_3{}^-$ in water	1.6×10^{-5}	cm^2 s^{-1}	[45]
$\overline{D}_{NO_3}$	Diffusion coefficient of $NO_3{}^-$ inside particles	$f_{O_2} \times D_{NO_3}$	cm^2 s^{-1}	–
D_M	Diffusion coeff. of monomers	0.6×10^{-5}	cm^2 s^{-1}	[29]
ε_m	Diffusivity of cell membrane layers relative to water	7.9×10^{-4}	–	[31]
r_C	Radius of cellular cytoplasm	0.27	μm	Calculated

L_{m}	Thickness of cell membrane layer	8×10^{-3}	µm	[46]
$\rho_{\mathrm{CN},B}$	Bacterial C:N	3.7	µg C µg N^{-1}	[47]
m_B	Mortality rate	0.1	d^{-1}	[40]
x_B	Mass of bacteria	5×10^{-8}	µg C	[15]
r_B	Radius of bacteria	0.29	µm	Eq. (S1)
V	Volume of bacteria	0.1058	μm^{3}	Eq. (S1)
r_P	Radius of particle	variable	cm	[9]
ρ	Fraction of diazotrophs compared to total bacteria	0.01	–	–
n_0	Constant for particle number	3.5×10^{-4}	# particle $cm^{\xi-4}$	[37]
ξ	Number spectral slope	3	–	[37]
T_{ref}	Reference temperature	20	^{0}C	[23]
$Q_{10,h}$	Q_{10} value for hydrolysis	2	–	[34]
$Q_{10,A}$	Q_{10} value for affinity	1.5	–	[35]
$Q_{10,R}$	Q_{10} value for respiration	2	–	[36]

References

1. Azúa, I., Unanue, M., Ayo, B., Artolozaga, I. & Iriberri, J. Influence of age of aggregates and prokaryotic abundance on glucose and leucine uptake by heterotrophic marine prokaryotes. *Int. Microbiol.* **10**, 13–18 (2007).
2. Ploug, H. Small-scale oxygen fluxes and remineralization in sinking aggregates. *Limnol. Oceanogr.* **46**, 1624–1631 (2001).
3. Lopez-Fernandez, P. *et al.* Bioavailability of sinking organic matter in the Blanes canyon and the adjacent open slope (NW Mediterranean Sea). *Biogeosciences* **10**, 3405–3420 (2013).
4. Waldemar, S., Kiersztyn, B. & Chróst, R. J. The dynamics of protein decomposition in lakes of different trophic status - Reflections on the assessment of the real proteolytic activity In Situ. *J. Microbiol. Biotechnol.* **17**, 897–904 (2007).
5. Piontek, J. *et al.* The utilization of polysaccharides by heterotrophic bacterioplankton in the Bay of Biscay (North Atlantic Ocean). *J. Plankton Res.* **33**, 1719–1735 (2011).
6. Passow, U. & Alldredge, A. L. Distribution, size and bacterial colonization of transparent exopolymer particles (TEP) in the ocean. *Mar. Ecol. Prog. Ser.* **113**, 185–198 (1994).
7. Geisler, E., Bogler, A., Rahav, E. & Bar-Zeev, E. Direct Detection of Heterotrophic Diazotrophs Associated with Planktonic Aggregates. *Sci. Rep.* **9**, 1–9 (2019).
8. Vaccaro, R. F., Hicks, S. E., Jannasch, H. W. & Carey, F. G. the Occurrence and Role of Glucose in Seawater. *Limnol. Oceanogr.* **13**, 356–360 (1968).
9. Bianchi, D., Weber, T. S., Kiko, R. & Deutsch, C. Global niche of marine anaerobic metabolisms expanded by particle microenvironments. *Nat. Geosci.* **11**, 1–6 (2018).
10. Millero, F. J. *Chemical Oceanography*. (CRC Press, 2005).
11. Paerl, R. W., Hansen, T. N. G., Henriksen, N. N. S. E., Olesen, A. K. & Riemann, L. N-fixation and related O2 constraints on model marine diazotroph Pseudomonas stutzeri BAL361. *Aquat. Microb. Ecol.* **81**, 125–136 (2018).
12. Paulmier, A., Ruiz-Pino, D. & Garçon, V. CO2 maximum in the oxygen minimum zone (OMZ). *Biogeosciences* **8**, 239–252 (2011).
13. Lee, C. & Cronin, C. The vertical flux of particulate organic nitrogen in the sea: decomposition of amino acids in the Peru upwelling area and the equatorial Atlantic. *J. Mar. Res.* **40**, 227–251 (1982).
14. Romanova, N. D. & Sazhin, A. F. Relationships between the cell volume and the carbon content of bacteria. *Oceanology* **50**, 522–530 (2010).
15. Simon, M., Alldredge, A. & Azam, F. Bacterial carbon dynamics on marine snow. *Mar. Ecol. Prog. Ser.* **65**, 205–211 (1990).
16. Yoshida, T., Hairston, N. G. & Ellner, S. P. Evolutionary trade-off between defence against grazing and competitive ability in a simple unicellular alga, Chlorella vulgaris. *Proceedings. Biol. Sci.* **271**, 1947–1953 (2004).
17. Ploug, H., Grossart, H. P., Azam, F. & Jørgensen, B. B. Photosynthesis, respiration, and carbon turnover in sinking marine snow from surface waters of Southern California Bight: Implications for the carbon cycle in the ocean. *Mar. Ecol. Prog. Ser.* **179**, 1–11 (1999).

18. Huston, A. L. & Deming, J. W. Relationships between microbial extracellular enzymatic activity and suspended and sinking particulate organic matter: Seasonal transformations in the North Water. *Deep. Res. Part II Top. Stud. Oceanogr.* **49**, 5211–5225 (2002).

19. Ayo, B. *et al.* Kinetics of glucose and amino acid uptake by attached and free-living marine bacteria in oligotrophic waters. *Mar. Biol.* **138**, 1071–1076 (2001).

20. Fouilland, E., Gosselin, M., Rivkin, R. B., Vasseur, C. & Mostajir, B. Nitrogen uptake by heterotrophic bacteria and phytoplankton in Arctic surface waters. *J. Plankton Res.* **29**, 369–376 (2007).

21. Thingstad, T. Simulating the response to phosphate additions in the oligotrophic eastern Mediterranean using an idealized four-member microbial food web model. *Deep. Res. Part II Top. Stud. Oceanogr.* **52**, 3074–3089 (2005).

22. Kondo, R., Nedwell, D. B., Purdy, K. J. & de Queiroz Silva, S. Detection and enumeration of sulphate-reducing bacteria in estuarine sediments by competitive PCR. *Geomicrobiol. J.* **21**, 145–157 (2004).

23. Bentzon-Tilia, M., Severin, I., Hansen, L. H. & Riemann, L. Genomics and ecophysiology of heterotrophic nitrogen-fixing bacteria isolated from estuarine surface water. *MBio* **6**, (2015).

24. Capone, D., Bronk, D., Mulholland, M. & Carpenter, E. *Nitrogen in the marine environment*. (Academic press, 2008).

25. Mislan, K. A. S., Stock, C. A., Dunne, J. P. & Sarmiento, J. L. Group behavior among model bacteria influences particulate carbon remineralization depths. *J. Mar. Res.* **72**, 183–218 (2014).

26. Flynn, K. J. Incorporating plankton respiration in models of aquatic ecosystem function. in *Respiration in aquatic ecosystems* (eds. del Giorgio, P. A. & Williams, LeB, P. J.) 248–266 (Oxford University Press, 2005).

27. Großkopf, T. & LaRoche, J. Direct and indirect costs of dinitrogen fixation in Crocosphaera watsonii WH8501 and possible implications for the nitrogen cycle. *Front. Microbiol.* **3**, 236 (2012).

28. Goldman, E. & Green, L. *Practical handbook of microbiology*. (CRC Press, Taylor & Francis Group, 2008).

29. Stein, W. D. *Channels, Carriers, and Pumps: An Introduction to Membrane Transport*. (Academic press, 1990).

30. Ploug, H. & Passow, U. Direct measurement of diffusivity within diatom aggregates containing transparent exopolymer particles. *Limnol. Oceanogr.* **52**, 1–6 (2007).

31. Inomura, K., Bragg, J. & Follows, M. J. A quantitative analysis of the direct and indirect costs of nitrogen fixation: A model based on Azotobacter vinelandii. *ISME J.* **11**, 166–175 (2017).

32. Garcia, H. *et al. World Ocean Atlas 2018, Volume 4: Dissolved Inorganic Nutrients (phosphate, nitrate and nitrate+nitrite, silicate). A. Mishonov Technical Ed.; NOAA Atlas NESDIS 84*. (2019).

33. Garcia, H. *et al. World Ocean Atlas 2018, Volume 3: Dissolved Oxygen, Apparent Oxygen Utilization, and Oxygen Saturation. A. Mishonov Technical Ed.; NOAA Atlas NESDIS 83*. (2019).

34. Li, Y. *et al.* Extracellular Enzyme Activity and Its Implications for Organic Matter Cycling in Northern Chinese Marginal Seas. *Front. Microbiol.* **10**, 1–13 (2019).

35. Serra-Pompei, C., Hagstrom, G. I., Visser, A. W. & Andersen, K. H. Resource limitation determines temperature response of unicellular plankton communities. *Limnol. Oceanogr.* **64**, 1627–1640

(2019).

36. Eppley R, W. Temperature and Phytoplankton Growth in the Sea. *Fish. Bull.* **70**, 1063–1085 (1972).

37. Jackson, G. A. *et al.* Particle size spectra between 1 μm and 1 cm at Monterey Bay determined using multiple instruments. *Deep. Res. Part I Oceanogr. Res. Pap.* **44**, 1739–1767 (1997).

38. Lee, C. & Bada, J. L. Dissolved amino acids in the equatorial Pacific, the Sargasso Sea, and Biscayne Bay. *Limnol. Oceanogr.* **22**, 502–510 (1977).

39. Wright, J. & Colling, A. The Seawater Solution. in *Seawater: its Composition, Properties and Behaviour* 85–127 (El, 1995).

40. Billen, G. & Becquevort, S. Phytoplankton-bacteria relationship in the Antarctic marine ecosystem. *Polar Res.* **10**, 245–254 (1991).

41. Treude, T. *et al.* Anaerobic oxidation of methane and sulfate reduction along the Chilean continental margin. *Geochim. Cosmochim. Acta* **69**, 2767–2779 (2005).

42. Paulmier, A., Kriest, I. & Oschlies, A. Stoichiometries of remineralisation and denitrification in global biogeochemical ocean models. *Biogeosciences* **6**, 923–935 (2009).

43. Henze, M., van Loosdrecht, M. C. M., Ekama, G. A. & Brdjanovic, D. *Biological wastewater treatment principles, modelling and design*. (IWA Publishing, 2008).

44. McCabe, M. & Laurent, T. C. edge of the respiration rates of the aortal walls it is possible to calculate transport coefficients for oxygen through the wall of the artery . Again b y such a method the results obtained are considerably lower than the values quoted for oxygen diffusio. *Biochim. Biophys. Acta* **399**, 131–138 (1975).

45. Yuan-Hui, L. & Gregory, S. Diffusion of ions in sea water and in deep-sea sediments. *Geochim. Cosmochim. Acta* **38**, 703–714 (1974).

46. Prescott, L., Harley, J. & Klein, D. Procaryotic Cell Structure and Function. in *Microbiology* 41–73 (McGrawn Hill, 2002).

47. Lee, S. & Fuhrman, J. A. Relationships between Biovolume and Biomass of Naturally Derived Marine Bacterioplankton. *Appl. Environ. Microbiol.* **53**, 1298–303 (1987).
